# Prevalence of Hyperhomocysteinemia in China: A Systematic Review and Meta-Analysis

**DOI:** 10.3390/nu7010074

**Published:** 2014-12-29

**Authors:** Boyi Yang, Shujun Fan, Xueyuan Zhi, Yinuo Wang, Yanxun Wang, Quanmei Zheng, Guifan Sun

**Affiliations:** 1 Research Center of Environment and Non-Communicable Disease, School of Public Health, China Medical University, Shenyang 110013, China; E-Mails: boyiyangcmu@163.com (B.Y.); fanfan0721ykl@163.com (S.F.); zhixy90smile@126.com (X.Z.); qmzheng@mail.cmu.edu.cn (Q.Z.); 2 Division of Molecular Preventive Medicine, Shanghai Institute of Targeted Therapy and Molecular Medicine, Shanghai 200433, China; E-Mails: 13817895706@163.com (Y.W.); wangyanxun@genechina.com (Y.W.)

**Keywords:** hyperhomocysteinemia, prevalence, China, meta-analysis

## Abstract

Hyperhomocysteinemia (HHcy, total homocysteine concentrations > 15 μmol/L) has been associated with increased risk of many diseases. A systematic review was performed to summarize the prevalence of HHcy in China. We searched multiple international and Chinese scientific databases for relevant literature, and further manually screened reference lists and corresponded with original authors. Pooled prevalence of HHcy was calculated using random effects model. Subgroup analysis, meta-regression and sensitivity analysis were also performed. A total of 36 studies consisting 60,754 subjects (57.3% male; age range, 3–97 years) were finally included. The overall pooled prevalence of HHcy was 27.5%. Geographically, the prevalence was high in north areas, intermediate in central areas, and low in south areas, and was higher in inland *versus* coastal areas. The prevalence increased with age and was significantly higher in men than in women. Rural residents had a slightly higher HHcy prevalence than urban residents, and the studies conducted during 2006 to 2012 presented a higher HHcy prevalence than those during 1990 to 2005. In summary, the prevalence of HHcy in China is high, particularly in northern populations, the inlanders, males, and the elderly. Homocysteine-lowering strategies are necessary to reduce this highly preventable disorder.

## 1. Introduction

Hyperhomocysteinemia (HHcy), a pathological condition characterized by elevated total homocysteine (tHcy) concentrations (usually defined as tHcy concentrations > 15 μmol/L) in blood, is a well established risk factor for neural tube defects (NTDs), and it also has been associated with many non-communicable diseases (NCDs), including cardiovascular and cerebrovascular diseases, type 2 diabetes, and cancers [1,2,3,4,5]. In the past two decades, numerous studies have been conducted in developed countries to explore tHcy status and its determinants. The results of these studies suggest that HHcy could be caused by many factors such as genetic variations, environmental exposures, lifestyle habits, disease states, hormonal factors, and several drugs [6,7,8], and the prevalence of HHcy varied widely with geography, age, sex, and ethnicity. For example, the Hordaland Homocysteine Study demonstrated that tHcy concentrations were higher in men and the elderly [9]; Carmel and coworkers reported the lowest Hcy concentrations among Asian Americans, intermediate concentrations among Hispanic Americans, and the highest concentrations among Caucasians [10]; Amouzou and colleagues observed that people inhabiting coastal West Africa had a higher prevalence of HHcy than the inlanders [11].

Due to high geographical, ethnical, social, and dietary diversity in China, the risk factors for HHcy vary among different populations [12,13,14]. Thus, there might exist a varied prevalence of HHcy among populations residing in different regions. Additionally, along with the changing lifestyles and dietary habits resulted from recent rapid industrialization and economic development, NCDs have become the most prominent threat to Chinese people’s health [15]. Furthermore, although the prevalence of NTDs in China shows a downward trend in recent years because of periconceptional folic acid supplementation, it remains as high as 14.0 per 10,000 births, which means nearly 20,000 affected infants are born each year [16,17]. And northern rural areas still have the highest prevalence of NTDs in the world [17]. Considering HHcy is one of the risk factors for NTDs and many NCDs, having an insight on its prevalence is therefore essential as it can provide evidence-based information for the development of effective programs and strategies to prevent and control these diseases.

In recent years, researchers have conducted a number of epidemiological studies to investigate the prevalence of HHcy in China. These studies, however, were limited by a focus on the prevalence at regional levels, and the national prevalence of HHcy or the seriousness of the disorder remains unknown. We therefore conducted this meta-analysis to systematically review the prevalence of HHcy in China. In addition to estimating the overall prevalence of HHcy, we compared the prevalence rates across geographical region, age, gender, study setting, sample size, and year of data collection. Furthermore, we conducted a meta-regression to explore the impact of the above methodological factors and study population characteristics on prevalence estimates. 

## 2. Experimental Section

### 2.1. Literature Searches

We performed a systematic literature search in three English (Pubmed, Embase and Web of Science) and four Chinese (CBM, CNIK, Wanfang and VIP) databases for studies containing the data on the prevalence of HHcy among Chinese population, using a combined search strategy that included the following search terms: “homocysteine”, “hyperhomocysteinemia”, “prevalence”, “incidence”, “epidemiology”, “survey”, “China”, and “Chinese”. No language restriction was applied and the databases were searched from their inception to April 2014. We manually screened the reference lists of retrieved articles, and efforts were also made to obtain the additional data by contacting original authors.

### 2.2. Study Selection and Inclusion Criteria

**Figure 1:**
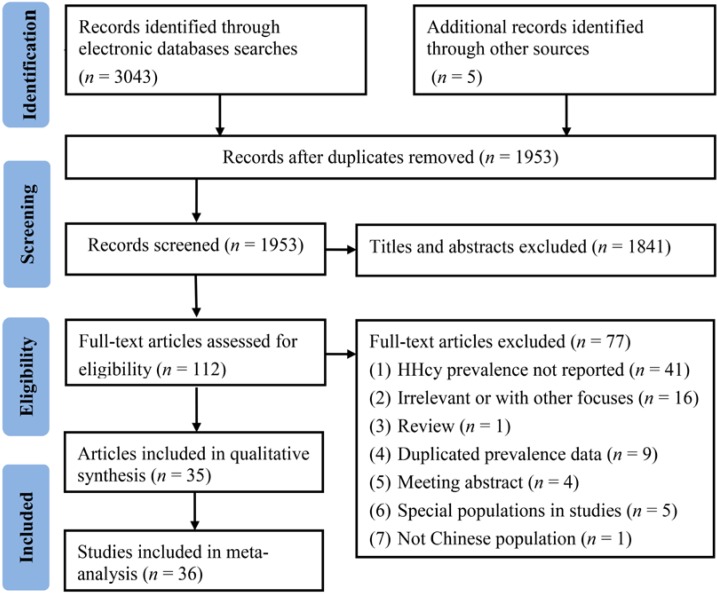
Flow diagram of study selection process in the meta-analysis.

After removing duplicates, the relevant articles were selected in two phases. In the first phase, titles and abstracts of articles were screened to identify potential studies. In the second phase, the full texts of identified articles were explored deeply to select the studies met the below mentioned inclusion criteria (Figure 1). Both screening phases were performed by two independent reviewers. Any discrepancies were reconciled through consensus or referred to a third reviewer as necessary.

Qualified studies had to meet the following criteria: (1) cross-sectional design, or baseline cross-sectional data from a cohort study or a trial; (2) provided data on the total number of participants and the number of HHcy participants, or reported the percentage of HHcy from which the number of HHcy participants could be calculated, or did not reported necessary data for pooling but they could be obtained by contacting the original authors; (3) the sample size was greater than 50; (4) if there are several articles based on the same population, only the one that provided the most detailed data was selected.

### 2.3. Data Extraction

Two of the reviewers independently extracted the following information from each included study: the first author’s name, publication year, gender proportion, year of data collection, age, province or municipality name, study region (north: Beijing, Tianjin, Shandong, Qinghai, Henan, Shanxi, Shaanxi, Xinjiang, Jilin, and Inner Mongolia; central: Shanghai, Chongqing, Jiangsu, Zhejiang, Hubei, and Sichuan; south: Guangdong, Hainan, and Taiwan), study location (coastal: Shanghai, Jiangsu, Guangdong, Tianjin, Shandong, Taiwan, Zhejiang, and Hainan; inland: Beijing, Qinghai, Henan, Shanxi, Shaanxi, Xinjiang, Jilin, Inner Mongolia, Chongqing, Hubei, and Sichuan), study setting (urban and rural), method of Hcy measurement, sample size, number of HHcy participants, total and age-, gender-, area-stratified prevalence of HHcy, and diagnostic criteria. A third reviewer confirmed all the extracted data. Missing raw data were requested from original authors by email.

### 2.4. Statistical Analysis

We performed meta-analyses using STATA package version 12.1 program (StataCorp, College Station, TX, USA). All statistical tests performed in this study were two tailed, and *P* value less than 0.05 was taken as statistically significant, unless otherwise stated. We calculated the point prevalence of HHcy and 95% confidence interval (CI) for each study, and then generated pooled prevalence estimates and 95% CI using the inverse variance method. Study heterogeneity was assessed using I^2^ statistics, with thresholds of ≥25%, ≥50%, and ≥75% correspond to low, moderate, and high heterogeneity, respectively [18]. The DerSimonian and Laird random effects model was adopted for summary statistics taking into account high heterogeneity among the included studies [19]. Subgroup analyses based on study region (north, central, and south), study location (inland and coastal), gender (men and women), age (<45, 45–65, and >65 years), sample size (<1000 and ≥1000), study setting (rural and urban), and year of data collection (1990–2005 and 2006–2012) were also performed. Furthermore, meta-regression was performed to explore whether the sources of the heterogeneity can be explained by some of methodological factors and study population characteristics, and to assess the association between these variables and the prevalence estimates [20]. Firstly, all the variables were tested separately in univariate analysis. Those with *p* < 0.2 in the univariate analysis were selected for inclusion in multivariate models [21]. Sensitivity analysis also was performed to examine the influence of excluding some specific studies on the overall estimates [22]. Finally, potential publication bias was tested using funnel plot and Egger’s regression intercept test [23].

## 3. Results

### 3.1. Study Selection Procedure and Characteristics

**Figure 2:**
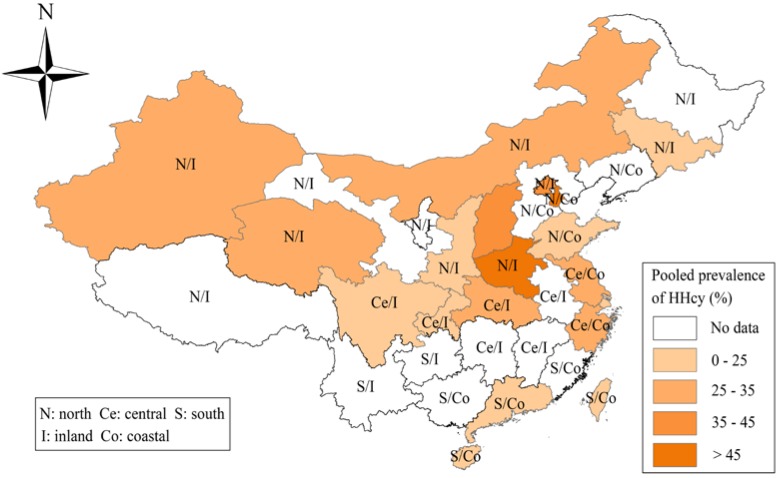
Provincial distribution pattern of pooled prevalence of hyperhomocysteinemia in China.

The process of identifying eligible epidemiological studies is summarized in Figure 1. A total of 3048 articles were identified in the initial search. After primary screening of titles and abstracts, 112 articles remained, and following full text review, 80 articles were further excluded. Although three of the excluded studies did not provide the raw data to estimate pooled prevalence and 95% CI, we obtained these data by emailing the original authors (Supplementary Table S1). Finally, 35 articles (eight published in English and 27 in Chinese) comprising 36 studies were included in the meta-analysis (Supplementary Table S1). The main characteristics of the included studies are shown in Table S1. The studies covered 16 provinces and three municipalities (Figure 2). Twenty studies were conducted in northern China, eight in central areas, seven in southern areas, and two in both northern and central areas (Figure 2). Twenty six studies included samples of urban population, five studies included samples of rural population and the remaining five studies included both. The method used for Hcy determination included high-performance liquid chromatography, enzyme-linked immunosorbent assay, fluorescence polarization immunoassay, fluorescence ration biochemical assay, enzyme transition method, enzymatic cycling assay, chemiluminescence immumoassay, and immunoturbidimetric assay. In terms of diagnostic criteria for HHcy, a cut-off value of 15 μmol/L was adopted by 32 studies, and other cut-off values such as 10, 11, 12.5, and 16 μmol/L, were adopted by the remaining four studies.

### 3.2. Meta-Analysis and Pooled Prevalence of HHcy

**Figure 3:**
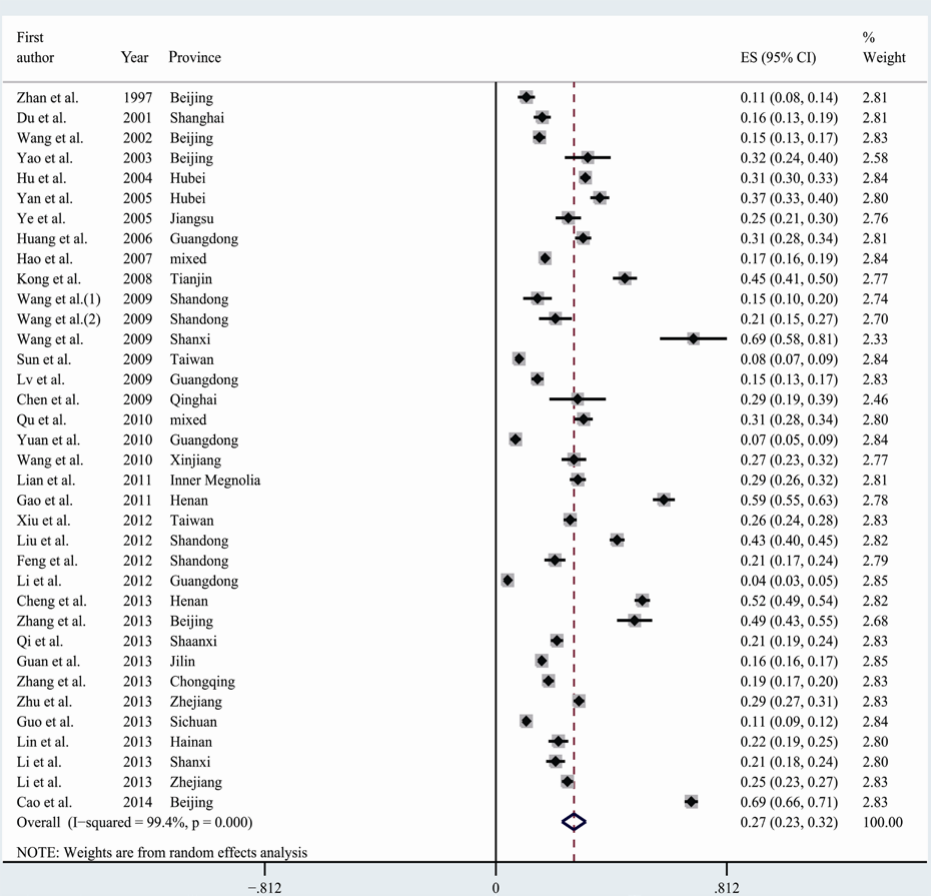
Forest plot of the prevalence of hyperhomocysteinemia in China.

Table 1 summarizes the overall and stratified pooled prevalence of HHcy by study characteristics such as geographical area, age, gender, year of data collection, sample size, and study setting. The overall pooled prevalence of HHcy in China was 27.5% with evidence of high heterogeneity (I^2^ = 99.4%, *p* < 0.001) (Table 1 and Figure 3). Stratified analyses based on geographical area showed that the HHcy prevalence was high in north areas (34.3%), intermediate in central areas (21.0%), and low in south areas (16.0%). The inlanders (31.5%) had a higher prevalence of HHcy than those living in coastal areas (23.0%). In three age groups, the prevalence was lowest in people aged <45 years (17.9%), intermediate in those aged 45–65 years (22.7%), and highest in those aged >65 years (35.2%). The prevalence for men (34.8%) was nearly twice higher than that for women (18.7%). Studies taking place in 1990–2005 had a lower prevalence than those in 2006–2012 (22.7% *vs.* 29.6%). The prevalence for the studies with sample size less than 1000 individuals was 29.5%, while for those with sample size over 1000 subjects, it decreased to 25.4%. Population residing in rural areas had a slightly higher HHcy prevalence than those residing in urban areas (28.1% *vs.* 26.5%). Test for heterogeneity was significant in all the subgroups (Table 1).

### 3.3. Sensitivity and Meta-Regression Analyses

To confirm the stability and liability of the meta-analysis, sensitivity analysis was performed. The prevalence estimates were not materially changed by excluding four studies using other cut-off values (10, 11, 12.5 and 16 μmol/L) for HHcy; excluding three studies with sample size <100 or >20,000; excluding four studies that did not report gender proportion; excluding five studies that did not report year of data collection; excluding three studies that did not report Hcy measurement method; and excluding 11 studies that did not report gender proportion, year of date collection, study setting, and Hcy measurement method (Table 2). These findings indicate that the results of our meta-analysis were robust. Additionally, meta-regression was employed to explore potential sources of heterogeneity. Information on mean age was not reported in nearly half of the studies, thus this variable was not included in the meta-regression model. Finally, the meta-regression was restricted to 28 studies that provided full information on publication year, gender proportion, sample size, study region, year of data collection, study setting, diagnostic criteria, and method for HHcy measurement. As summarized in Table 3, the aforementioned nine variables were primarily examined in the univariate meta-regression. Three variables publication year, study location, and study region were then selected in the multivariate meta-regression; however, none of these variables was significantly associated with the detected heterogeneity.

### 3.4. Publication Bias

**Figure 4:**
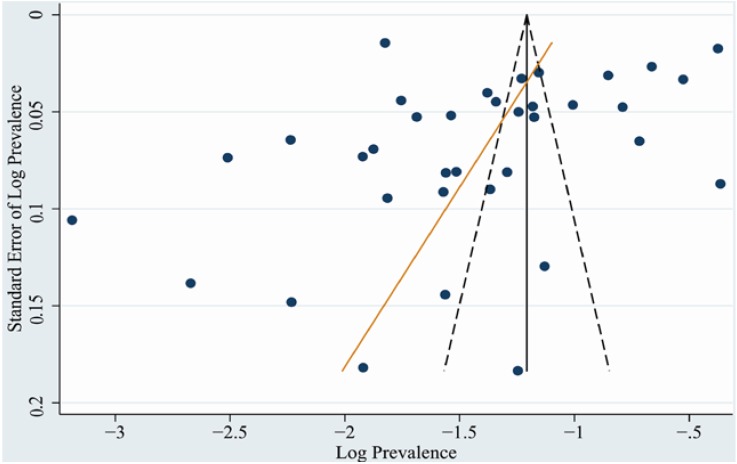
Funnel plot of 36 studies included in the meta-analysis.

The shape of funnel plot did not reveal obvious asymmetry (Figure 4). The results of Egger’s test also showed little evidence of significant publication bias among the contributing studies (*p* = 0.198).

**Table 1:** Stratified meta-analyses of the prevalence of HHcy in China.

Subgroup	No. of Studies	No. of Total Participants	No. of Cases	Prevalence (%)	95% CI	Heterogeneity Test	
						I^2^ (%)	*p* Value
Total	36	60,754	13,183	27.5	(23.3–31.6)	99.4	<0.001
Study Region							
North	21	37,678	8780	34.3	(26.7–42.0)	99.5	<0.001
Central	10	13,588	3020	21.0	14.5–27.5	99.0	<0.001
South	7	9080	1276	16.0	(9.4–22.6)	99.1	<0.001
Study Location							
Inland	19	42,703	9096	31.5	(25.0–38.0)	99.5	<0.001
Coastal	15	14,777	3411	23.0	(16.0–29.9)	99.3	<0.001
Sex							
Men	19	27,982	7736	34.8	(29.1–40.5)	98.8	<0.001
Women	20	19,700	2120	18.7	(15.2–22.2)	98.0	<0.001
Age							
<45	10	20,333	3062	17.9	(15.5–20.4)	94.5	<0.001
45–65	9	9164	1846	22.7	(19.0–26.5)	93.7	<0.001
>65	8	5039	974	35.2	(22.0–48.4)	99.2	<0.001
Sample Size							
<1000	19	8770	2475	29.5	(22.8–36.1)	98.3	<0.001
≥1000	17	51,984	10,708	25.4	(19.4–31.3)	99.7	<0.001
Year of Data Collection							
1990–2005	14	14,501	2690	22.7	(16.8–28.5)	99.1	<0.001
2006–2012	20	44,700	9938	29.6	(23.4–35.8)	99.5	<0.001
Study Setting							
Rural	8	4650	1196	28.1	(21.1–35.1)	96.6	<0.001
Urban	30	55,633	12,042	26.5	(21.7–31.3)	99.5	<0.001

HHcy, hyperhomocysteinemia; No, number; CI, confidence interval.

**Table 2:** Sensitivity analyses of pooled prevalence of HHcy in China.

Models	Available Studies for Analysis	Prevalence (%) (95% CI)	Heterogeneity Test	
			I^2 ^(%)	*p* Value
1. Exclude studies that cut-point for HHcy was not 15 μmol/L	32	27.4 (23.0–31.8)	99.5	<0.001
2. Exclude studies with sample size <100 or >20,000	33	26.7 (21.5–31.9)	99.5	<0.001
3. Exclude studies that gender proportion was not reported	32	28.1 (23.8–32.4)	99.3	<0.001
4. Exclude studies that year of data collection was not reported	31	26.6 (22.1–31.0)	99.5	<0.001
5. Exclude studies that Hcy Measurement method was not reported	33	28.6 (24.2–32.9)	99.3	<0.001
6. Exclude studies that gender proportion, year of date collection, study setting, and Hcy measurement method was not reported	25	27.6 (22.7–32.5)	99.4	<0.001

HHcy, hyperhomocysteinemia; Hcy, homocysteine; CI, confidence inteval.

**Table 3:** Univariate and multivariate meta-regression for prevalence of HHcy.

Variables	No. of study (*n* = 28)	Coefficient (95% CI)	*P* value
Univariate meta-regression			
**Year of publication**	28	0.03 (−0.03–0.08)	0.288
**Male% in sample**	28	0.001 (−0.01–0.02)	0.862
**Sample size**	28	0.00 (−0.00–0.00)	0.435
**Study location**			
Inland	19	Reference	
Coastal	9	−0.35 (−0.84–0.14)	0.153
**Study region**			0.054
South	5	Reference	
Central	6	0.54 (−0.15–1.22)	0.119
North	17	0.71 (0.14–1.30)	0.017
**Study period**			
2006–2012	18	Reference	
1990–2005	10	−0.11 (−0.60–0.38)	0.656
**Study setting**			0.451
Rural and urban	4	Reference	
Urban	21	0.39 (−0.29–1.06)	0.251
Rural	3	0.51 (−0.44–1.47)	0.278
**Diagnostic criteria**			
15 μmol/L	25	Reference	
Others	3	0.16 (−0.60–0.92)	0.671
**Methods of Hcy measurement**			0.798
FPIA	5	Reference	
ECA	9	−0.10 (−0.81–0.62)	0.783
HPLC	8	0.07 (−0.66–0.80)	0.846
Others	6	0.22 (−0.55–1.00)	0.555
Multivariate meta-regression			0.0511
**Year of publication**	28	0.04 (−0.01–0.09)	0.242
**Study location**			
Inland	19	Reference	
Coastal	9	−0.16 (−0.70–0.38)	0.614
**Study region**			0.294
South	5	Reference	
Central	6	0.48 (−0.25–1.21)	0.185
North	17	0.66 (−0.01–1.33)	0.052

HHcy, hyperhomocysteinemia; No, number; CI, confidence interval; FPIA, fluorescence polarization immunoassay; ECA, enzymatic cycling assay; HPLC, high-performance liquid chromatography.

## 4. Discussion

This meta-analysis based on 60,754 subjects derived from 36 studies covering 19 provinces and municipalities in China, enabling us to assess reliable prevalence estimates of HHcy at the national level. To our knowledge, this is the first meta-analysis of prevalence of HHcy in China, and the results showed that the overall estimate of HHcy prevalence was 27.5%. The pooled prevalence is similar to that reported in Brazil [24] and Lebanon [25], but higher than that reported in Switzerland [26], Costa Rica [27], and Korea [28], and lower than that reported in Iran [29], Algeria [30], and coastal West Africa [11]. The different prevalence rates reported in various countries could be due to different population inclusion criteria, genetic backgrounds, as well as differences in HHcy risk factor profiles (such as nutrition status, environment exposures, and lifestyles) in the population of each country. It is noteworthy that the prevalence of HHcy has been decreasing in some developed countries because they had established policies for folic acid fortification of cereals and flour to prevent NTDs. For example, before folic acid fortification, the prevalence of HHcy was 18.7% and 29% in North America [31] and Australia [32] (the cut-off value was determined as 13 μmol/L in the two studies), respectively. However, after the fortification was implemented, the prevalence decreased to 9.8% and 10% in North America and Australia, respectively [31,32]. More importantly, the potential benefit of Hcy-lowering with B vitamins has been demonstrated in many studies [33,34,35,36,37,38,39,40,41,42]. A cohort study by Berry R.J. and collaborators showed that daily periconceptional intake of 400 μg of folic acid successfully prevented 79% and 41% of NTDs in northern and southern regions of China, respectively [33]. Additionally, several cohort, prospective, and retrospective studies and meta-analyses of recent, large, randomized vitamin trials support the beneficial effect of Hcy-lowering in cardiovascular prevention, especially in the primary prevention [34,35,36,37]. One of the main concerns of folic acid supplementation, particularly the population-based exposure to folic acid through fortification, is that it may increase the risk of cancer [38]. However, a meta-analysis of 15 randomized controlled trials with 50,144 individuals found that folic acid fortification had no significant effect on total cancer incidence, but reduced the risk of melanoma [39]. Several recent meta-analyses also reported that higher dietary folate intake could significantly reduce the risk of breast cancer, pancreatic cancer, and lung cancer [40,41,42]. The example mentioned above indicate that HHcy is highly preventable and effective Hcy-lowering strategies are needed in China, especially considering its relatively high prevalence and strong associations with NTDs and some NCDs that in China have become the major public health concerns in recent years [15,16,17].

Stratified analysis by geographical area showed that the prevalence of HHcy seemed to increase in a roughly northerly direction. However, these results should be interpreted with caution because only seven studies covering three provinces were conducted in the south region. The limited number of studies and sample size might have compromised our estimates. Additional studies, therefore, are needed to explore the prevalence of HHcy in the remaining un-surveyed areas and to confirm whether there is a real geographical gradient in HHcy prevalence. Another important finding was that the Chinese inlanders had higher prevalence of HHcy than coastal residents, which is consistent with the results from a previous study conducted among Chinese hypertensive adults [43], but is contrary to findings from a small study in coastal West Africa [11]. Many factors may contribute to these geographical disparities found in our study. One of the main factors may be different dietary habits in these regions. Folate and vitamin B_12_ intake, the most important nutritional determinant of HHcy, is lower in the diet of Chinese northerners than in that of the southerners [12]. Seafood (rich in betaine and vitamin B_12_) consumption in coastal regions is higher than that in inland regions [44,45]. Genetic background may be another essential factor. The *MTHFR* C677T polymorphism is the most common genetic determinants of HHcy especially under the conditions of low dietary folate. Our previous studies found that the frequencies of 677T allele and 677 TT genotype showed a decreasing trend from northern to southern China [13]. Wang *et al*. [14] reported that the 677T allele frequency was higher in coastal Chinese population and lower in inland Chinese population. Other factors such as physical activity, smoking, coffee, and environmental exposures may also be responsible. Therefore, further studies including genetic, nutritional, environmental, and demographic factors are required to explain the geographical distribution of HHcy.

Consistent with observations from some large population-based studies [9,46], the findings of our meta-analysis suggest that the prevalence of HHcy increased with age and was significantly higher in men than in women. Possible explanations for the sex-specific variation include differences in muscle mass, estrogen status, life styles, and vitamin status [47]. It has been confirmed that Hcy production is related to creatine-creatinine synthesis [48]. Males usually have more muscle mass and therefore greater demand for creatine biosynthesis, which consequently results in more Hcy production [49]. Hormonal differences between men and women could also contribute to the sex-related difference. A clinical study of cross-sex hormone administration to transsexuals showed that male-to-female transsexuals who were treated with ethinyl estradiol and antiandrogen for four months showed a significant decrease in Hcy levels. Conversely, female-to-male transsexuals who received androgen therapy resulted in increased plasma Hcy [50]. Another randomized controlled study found that estrogen replacement significantly lowered Hcy concentrations in postmenopausal women [51]. Additionally, compared with women, Chinese men have a higher prevalence of alcohol intake and cigarette smoking, which are all positively associated with Hcy concentrations [12]. Furthermore, folate, vitamin B_12_, and vitamin B_6_ status are different between the two sexes, which may also partly explain the gender differences [47]. Possible mechanisms involved in the age-related increase in HHcy prevalence include changes in renal function, decreases in vitamin levels, as well as impaired renal metabolism of Hcy [9,52,53]. Previous studies demonstrated a significant age-sex interaction such that the gender-difference narrows with age, which could be a consequence of an effect of estrogen on Hcy metabolism [46]. Most studies included in our review did not provided detailed data on age- and sex- specific prevalence of HHcy, thus precluding us to explore and confirm the age-sex interaction. Nevertheless, the elderly population of China has increased dramatically in recent years. Given that both older age and HHcy are important risk factors for several kinds of NCDs, public health programs should play an emphasize on reducing the high prevalence of HHcy among Chinese elderly populations.

The pooled prevalence of HHcy increased from 22.7% in 1990–2005 to 29.6% in 2006–2012, suggesting HHcy prevalence might experience an increase over the last two decades. This is probably due to changing lifestyles and dietary habits resulted from rapid industralization in China. However, limited by the number of studies included in the review, we could just compare the differences in HHcy prevalence between the two time periods but could not explore the continual change in the prevalence from 1990 to 2012. Longitudinal studies with the same survey design and methods over time are therefore needed to explore the variation of HHcy prevalence in different period. Individuals residing in rural areas had a slightly higher prevalence of HHcy than those living in urban areas, which is contrary to the findings from a previous study in China (Reference 9 in Supplementary Table S1). However, it is notable that only eight studies were conducted in rural areas while 30 studies were in urban areas, thus these results should be interpreted with great caution.

In interpreting the findings of the present meta-analysis, some limitations should be carefully considered. Firstly, significant heterogeneity between studies was observed. Although potential sources of heterogeneity, including publication year, gender proportion, sample size, geographical area, study period, study setting, diagnostic criteria, and method of Hcy measurement, were explored by subgroup analyses and meta-regression, none of them sufficiently explain the heterogeneity. These results indicate that other unmeasured characteristics in study population and limitations of the included studies likely influence the detected heterogeneity. Secondly, the included studies only cover 19 regions and the sample size of some regions is not large enough, which limits our ability to provide more precise estimates at the national level. Despite the shortcomings, our meta-analysis still has some clear advantages: (1) the review is based on a substantial number of participants, which guarantees the statistical power and precision of estimates; (2) as many of the studies included in our meta-analysis are described in Chinese, pooling such data and introducing them in this review may be of value to non-Chinese readers and future researches in Hcy and related fields; (3) including unpublished studies and contacting original investigators to obtain the missing data, thus reducing possible publication bias; (4) although there exist variations in population characteristics and methodological differences such us diagnostic criteria for HHcy, sensitivity analyses excluding specific studies showed that our results were statistically robust.

## 5. Conclusions

In summary, our study shows that the prevalence of HHcy in China is high, particularly in northern populations, the inlanders, males, and the elderly. Furthermore, the prevalence has increased in recent years. These findings indicate that HHcy has become an important public health issue in China, as the disorder is associated with increased risk of NTDs and many NCDs. Thus, there is an urgent need for policy makers and health care providers at national and regional levels to develop effective prevention and intervention programs (such as folic acid supplementation and lifestyle changes) to reduce this highly preventable disorder. Considering the limitations noted above, further well-designed, national, and population-based surveys on the prevalence of HHcy are warranted.

## Supplementary Files

Supplementary File 1
