# Supplementary material for: Prevalence of Hyperhomocysteinemia in China: A Systematic Review and Meta-Analysis
Source: Nutrients. 2014 Dec 29;7(1):74–90. doi: 10.3390/nu7010074 (PMC4303827; doi:10.3390/nu7010074)
Supplement: Supplementary File 1 [file nutrients-07-00074-s001.docx]

Supplementary Information

**Table S1.** Characteristics of the epidemiological studies on the prevalence of HHcy in China.

| **First Author,  Publication Year** | **Year of Data  Collection** | **Gender (Male %)** | **Age (Years)** | **Province; Area** | **Study Setting** | **Method of Hcy Measurement** | **Sample  Size** | **Prevalence  of HHcy (%)** | **Criteria for HHcy** |
| --- | --- | --- | --- | --- | --- | --- | --- | --- | --- |
| Zhan *et al*. 1997 [1] | 1990.1–1991.12 | 0 | NR | Beijing (N/I) | U and R | HPLC | 401 | 10.72 | >15 μmol/L |
| Du *et al*. 2001 [2] | 1999 | NR | NR | Shanghai (Ce/Co) | U | ELISA | 589 | 16.30 | ≥15 μmol/L |
| Wang *et al*. 2002 [3] | 1999.9–1999.10 | 43.92 | 35–64 | Beijing (N/I) | U and R | FPIA | 1168 | 15.30 | >15 μmol/L |
| Yao *et al*. 2003 [4] | NR | 48.46 | 35–49 | Beijing (N/I) | U | HPLC | 130 | 25.38 | >11 μmol/L |
| Hu *et al*. 2004 [5] | NR | 51.97 | 13–83 | Hubei (Ce/I) | U | HPLC | 2465 | 31.48 | >15 μmol/L |
| Yan *et al*. 2005 [6] | 2000.12–2002.6 | 43.09 | 62 ± 8 | Hubei (Ce/I) | U | HPLC | 810 | 36.54 | ≥15 μmol/L |
| Ye *et al*. 2005 [7] | NR | NR | 35–60 | Jiangsu (Ce/Co) | U | ELISA | 369 | 25.47 | ≥15 μmol/L |
| Huang *et al*. 2006 [8] | NR | 23.76 | NR | Guangdong (S/Co) | R | FRBA | 1020 | 30.69 | >15 μmol/L |
| Hao *et al*. 2007 [9] | 2001 | 48.60 | 35–64 | Mixed (N/C) | U and R | HPLC | 2464 | 17.29 | ≥16 μmol/L |
| Kong *et al*. 2008 [10] | NR | 42.90 | 55–94 | Tianjin (N/Co) | U | ETM | 698 | 45.40 | >15 μmol/L |
| Wang *et al*. 2009 [11] | 2003 | 50.00 | 46.2 ± 8.5 | Shandong (N/Co) | R | HPLC | 191 | 14.7 | >15 μmol/L |
| Wang *et al*. 2009 [11] | 2003 | 56.00 | 46.9 ± 8.6 | Shandong (N/Co) | R | HPLC | 191 | 20.9 | >15 μmol/L |
| Chen *et al*. 2009 [12] | 2008.9–2008.11 | 50.00 | 18–24 | Qinghai (N/I) | NR | FPIA | 80 | 30.7 | >15 μmol/L |
| Sun *et al*. (2009) [13] | 1994–1995 | NR | ≥21 | Taiwan (S/Co) | U | FPIA | 2117 | 8.12 | ≥15 μmol/L |
| Qu *et al*. 2010 [14] | 2006 | 49.75 | 65–74 | Mixed | R | HPLC | 810 | 28.75 | >15 μmol/L |
| Wang *et al*. 2009 [15] | 2004 | 0 | 18–45 | Shanxi (N/I) | R | FPIA | 59 | 69.50 | >15 μmol/L |
| Lv *et al*. 2009 [16] | 2008.8–2008.12 | 66.06 | 17–83 | Guangdong (S/Co) | U | HPLC | 1108 | 14.62 | >15 μmol/L |
| Yuan *et al*. 2010 [17] | 2009.1–2009.5 | 56.64 | 19–78 | Guangdong (S/Co) | U | ECA | 738 | 6.91 | ≥15 μmol/L |
| Wang *et al*. 2010 [18] | 2007.1–2008.3 | 46.08 | 49.4 ± 9.7 | Xinjiang (N/I) | U | ELISA | 408 | 27.45 | ≥15 μmol/L |
| Lian *et al*. 2011 [19] | 2009.1–2010.12 | 64.52 | ≥21 | Inner Mongolia (N/I) | U | ECA | 992 | 28.83 | >15 μmol/L |
| Li *et al*. 2012 [20] | 1999–2000 | NR | 65–97 | Guangdong (S/Co) | U | NR | 2135 | 4.12 | >15 μmol/L |
| Feng *et al*. 2012 [21] | 2012.2–2012.12 | 52.68 | ≥20 | Shandong (N/Co) | U | NR | 467 | 20.77 | >15 μmol/L |
| Liu *et al*. 2012 [22] | 2008.4–2010.9 | NR | 24–70 | Shandong (N/Co) | U | ETM | 1379 | 42.64 | >15 μmol/L |

**Table S1.** *Cont*

| **First Author,  Publication Year** | **Year of Data  Collection** | **Gender (Male %)** | **Age (Years)** | **Province; Area** | **Study Setting** | **Method of Hcy Measurement** | **Sample  Size** | **Prevalence  of HHcy (%)** | **Criteria for HHcy** |
| --- | --- | --- | --- | --- | --- | --- | --- | --- | --- |
| Xiu *et al*. 2012 [23] | 1999–2000 | 53.2 | ≥65 | Taiwan (S/Co) | U and R | FPIA | 1412 | 26.13 | >15 μmol/L |
| Cheng *et al*. 2013 [24] | 2012 | 71.14 | >40 | Henan (N/I) | U | ECA | 1320 | 51.52 | >15 μmol/L |
| Zhu *et al*. 2013 [25] | 2011.5–2012.5 | 54.40 | 20–80 | Zhejiang (Ce/Co) | U | ECA | 2252 | 29.22 | >10 μmol/L |
| Li *et al*. 2013 [26] | 2011.9–2012.8 | 55.03 | 30–79 | Shanxi (N/I) | U | NR | 576 | 21.01 | >15 μmol/L |
| Qi *et al*. 2013 [27] | 2009.5–2012.9 | 49.49 | 40–72 | Shannxi (N/I) | U | ECA | 1364 | 21.48 | >15 μmol/L |
| Zhang *et al*. 2013 [28] | 2007.9–2008.10 | 44.80 | 54.7 ± 12.9 | Beijing (N/I) | U | CLIA | 250 | 48.80 | ≥15 μmol/L |
| Guan *et al*. 2013 [29] | 2010.6–2012.9 | 64.35 | 20–72 | Jilin (N/I) | U | ECA | 24807 | 16.12 | >15 μmol/L |
| Lin *et al*.2013 [30] | 2011.9–2012.4 | 62.55 | 30–88 | Hainan (S/Co) | U | ECA | 550 | 22.00 | >12.5 μmol/L |
| Gao *et al*. 2011 [31] | 2009.3–2010.10 | 69.37 | 35–89 | Henan (N/I) | U | ECA | 626 | 59.11 | ≥15 μmol/L |
| Li *et al*. 2013 [32] | 2012.1–2012.12 | 64.62 | 20–88 | Zhejiang (Ce/Co) | U | ECA | 1843 | 25.18 | >15 μmol/L |
| Guo *et al*. 2013 [33] | 2011.7–2012.5 | 49.34 | 3–94 | Sichuan (Ce/I) | U | ECA | 2033 | 10.67 | ≥15 μmol/L |
| Zhang *et al*. 2013 [34] | 2011.1–2012.12 | 58.25 | 18-95 | Chongqing (Ce/I) | U and R | IA | 1600 | 18.50 | >15 μmol/L |
| Cao *et al*. 2014 [35] | 2007.9–2009.1 | 42.02 | 45–96 | Beijing (N/I) | U | HPLC | 1497 | 68.74 | ≥15 μmol/L |

HHcy, hyperhomocysteinemia; Hcy, homocysteine; NR, not reported; N, north; S, south; Ce, central; I, inland; Co, coastal; U, urban; R, rural; HPLC, high-performance liquid chromatography; ELISA, enzyme-linked immunosorbent assay; FPIA, fluorescence polarization immunoassay; FRBA, fluorescence ration biochemical assay; ETM, enzyme transition method; ECA, enzymatic cycling assay; CLIA, chemiluminescence immumoassay; IA, immunoturbidimetric assays.

**References**

1. Zhan, S.Y.; Hu, Y.H.; Li, L.M.; Lee, B.L.; Ng, M.V.; Ong, C.N. A correlation study on homocysteine metabolism in pregnant women and neural tube defects in urban and rural areas. *Chin. J. Prev. Med.* **1997**, *31*, 221–223.
2. Du, X.; Shen, L.H.; Wu, C.; Gong, Y.X.; Gu, Z.D.; Lin, Y.; Shen, B.B.; Shen, G. The influence of multivitamin supplement on the plasma homocysteine level. *Shanghai. J. Med. Lab. Sci*. **2001**, *16*, 203–205.
3. Wang, W.; Zhao, D.; Liu, J.; Liu, J.; Zhang, Z.L.; Liu, J.; Liu, S.; Lin, Z.; Wu, Z.S. The distribution of serum homocysteine and its associated factors in a population of 1168 subjects in Beijing area. *Chin. J. Epidemiol.* **2002**, *23*, 32–35.
4. Yao, M.; Lichtenstein, A.H.; Roberts, S.B.; Ma, G.; Gao, S.; Tucker, K.L.; McCrory, M.A. Relative influence of diet and physical activity on cardiovascular risk factors in urban Chinese adults. *Int. J. Obes. Metab. Disord.* **2003**, *27*, 920–932.
5. Hu, C.P.; Shao, J.M.; Yan, J.T.; Fan, Q.; Liu, Z.J.; Tian, C.; Wu, H.L.; Li, X.P.; Wang, D.W. Study on the distribution of serum homocysteine and on multi-stepwise of regression analysis the associated factors in the population of community areas in Wuhan. *Chin. J. Epidemiol*. **2004**, *25*, 945–948.
6. Yan, J.T.; Jiang, H.P.; Ye, H.; Wen, R.; Shao, J.M.; Wang, D.W. Relationship between plasma homocysteine and C-reactive protein levels. *Chin. Circ. J.* **2005**, *20*, 118–120.
7. Ye, D.S.; Li, Y.; Li, D.Y.; Qian, W.H.; Yu, H.; Xia, Y.; Zhang, C.Q. The epidemiological study of the relationship between plasma homocysteine and hypertension. *J. Clin. Cardiol. (China)* **2005**, *21*, 536–538
8. Huang, H.W.; Guo, M.H.; Huang, J.X.; Lin, R.J.; Zhang, Y.; Chen, Y.L. The analysis of plasma homocysteine among 1020 residents in community. *Chin. J. Epidemiol.* **2006**, *27*, 721–724.
9. Hao, L.; Ma, J.; Zhu, J.; Stampfer, M.J.; Tian, Y.H.; Willett, W.C.; Li, Z. High prevalence of hyperhomocysteinemia in Chinese adults is associated with low folate, vitamin B-12, and vitamin B-6 status. *J. Nutr.* **2007**, *137*, 407–413.
10. Kong, H.Y.; Jiang, Y.G.; Huang, C.Y.; Fang, H.T.; Liu, J.; Sun, C.; Fang, H.Y.; Pang, W.
    A survey on serum homocysteine levels and cognitive function of the middle aged and elderly persons in Tianjin city. *J. Hygie. Res.* **2008**, *37*, 677–679.
11. Wang, Y.J.; Zhang, L.; Moslehi, R.; Ma, J.L.; Pan, K.F.; Zhou, T.; Liu, W.D.; Brown, L.M.; Hu, Y.G.; Pee, D.; *et al*. Long-term garlic or micronutrient supplementation, but not anti-Helicobacter pylori therapy, increases serum folate or glutathione without affecting serum vitamin B-12 or homocysteine in a rural Chinese population. *J. Nutr*. **2009**, *139*, 106–112.
12. Chen, Y.; Mao, H.Q.; Yang, H.L.; Wang, S.L.; Xing, Y.H.; Liu, Y.; Guo, Y.H. Study on the levels and correlation of plasma homocysteine and plasma folate in healthy adults. *J. Qinghai. Med. Colleg*. **2009**, *30*, 121–123.
13. Sun, Y.; Chien, K.L.; Hsu, H.C.; Su, T.C.; Chen, M.F.; Lee, Y.T. Use of serum homocysteine to predict stroke, coronary heart disease and death in ethnic Chinese—12-year prospective cohort study. *Circ. J.* **2009**, *73*, 1423–1430.
14. Qu, Q.G.; Gao, J.J.; Liu, J.M. Prevalence of hyperhomocysteinaemia in a Chinese elderly population. *Public. Health. Nutr*. **2010**, *13*, 1974–1981.
15. Wang, B.J. Study of Dietary Factors and Effect of Nutritional Intervention in the Pilot Regions of the Project “Intervention Program on Birth Defects” in Shanxi Province. Master’s Thesis, Peking Union Medical College, Tsinghua University, Beijing, China, 2009.
16. Lv, S.W. Determinants of Serum Homocysteine Concentrations: A Cross-Sectional, Population Based Study in Guangzhou. Master’s Thesis, Sun Yat-sen University, Guangdong, China, 2009.
17. Yuan, M.Y.; Qiu, J.J.; Yue, F.; Liu, L.H.; Wu, C.H.; Li, H.M.; Chen, M.L. Plasma homocysteine levels in healthy adult subjects with different age and sex. *Chin. J. Pathophysiol.* **2010**, *26*,
    2226–2228.
18. Wang, H.; Wu, G.Z.; Zhang, Y.; Zhang, X.Y.; Chen, Y.L.; Al, S.K. Association of homocysteine and its metabolic enzyme genes polymorphisms with essential hypertension in Xinjiang Kazakhs. *J. Clin. Rehabil. Tissue. Engine. Res.* **2010**, *14*, 6247–6252.
19. Lian, L. The investigation and analysis of plasma homocysteine levels in 992 cases of physical examination population. *Lab. Med. Clin.* **2011**, *8*, 2333–2336.
20. Li, M.; Zhou, H.Q.; Wan, X.C. Study on the levels of homocysteine and blood lipids in physical examination population in Hangzhou city. *Chin. J. Health. Lab. Tech.* **2013**, *23*, 1579–1581.
21. Feng, C.N.; Zhong, L.; Wang, W.; Sui, X.; Wang, H.; Fan, C.M. A survey study on risk factors for hyperhomocysteinemia. *Chin. Prac. Med.* **2012**, *7*, 109–110
22. Liu, S.B. Associations of plasma homocysteine levels with metabolic syndrome and its components. *Guide. China. Med.* **2012**, *10*, 510–511.
23. Xiu, L.L.; Lee, M.S.; Wahlqvist, M.L.; Chen, C.Y.; Huang, Y.C.; Chen, K.J.; Li, D. Low and high homocysteine are associated with mortality independent of B group vitamins but interactive with cognitive status in a free-living elderly cohort. *Nutr. Res.* **2012**, *32*, 928–939.
24. Cheng, S.J. Study on the prevalence of plasma homocysteine levels and BMI in the middle aged and the elderly senior intellectual population. *Chin. Prac. Med.* **2013**, *8*, 107–108.
25. Zhu, J.Q.; Xiao, Y.F.; Qin, G.M. A epidemiological survey on hypertension with hyperhomocysteinemia in healthy population. *J. Radioimmunol.* **2013**, *26*, 216–217.
26. Li, J. Analysis on hyperhomocysteinemia in physical examination population. *Public. Med. Forum.* **2013**, *17*, 1805–1806.
27. Qi, H.Y.; Li, H.; Zhou, H.L.; Wei, H.M. Study on level of lipid, folate and homocysteine in
    middle-aged and the elderly population in Yan-an. *J. Mod. Lab. Med.* **2013**, *28*, 135–138.
28. Zhang, M.H.; Ye, P.; Luo, L.M.; Xiao, W.K.; Wu, H.M.; Liu, D.J.; Liu, G.S. The relationship between plasma homocysteine and pulse wave velocity in a community based healthy subject group. *Chin. Circ. J.* **2013**, *28*, 132–135.
29. Guan, B.J.; Ding, X.; Zhang, J. A survey on prevalence of hyperhomocysteinemia among 24807 physical examination population in Changchun. *Chin. J. Clin. Res.* **2013**, *26*, 307–308.
30. Lin, L.Z. Study on plasma homocysteine levels and its determinants among 550 physical examination population in Haikou. *Hainan Med.* **2013**, *24*, 867–868.
31. Gao, L.P.; Zhang, Y.H.; Zhang, H.Q. Association between homocysteine and hypertension.
    *Chin. J. Misdiagn*. **2011**, *11*, 8396–8397.
32. Li, Z.Y.; Jin, X.L.; Yuan, L.; Li, CJ. Risk factors for hyperhomocysteinemia. *Guangzhou. Med.* **2012**, *43*, 21–22.
33. Guo, T.K.; Guo, C.L.; Gan, L.; Li, J.G.; Guo, Y. Reference range of homocysteine. *Lab. Med. Clin.* **2013**, *10*, 872–873.
34. Zhang, Z.J.; Tang, M.S.; Zou, Y.B.; Xiao, J.; Jiang, S.D.; Li, C.B.; Zhang, J.X. Distribution of plasma homocysteine levels in adult residents of Ba’nan district of Chongqing city. *J. Chongqing. Univ. (Med. Sci.).* **2013**, *53*, 70–72.
35. Cao, R.H.; Bai, Y.Y.; Xu, R.Y.; Ye, P. Homocysteine is associated with plasma high-sensitivity cardiac troponin T levels in a community-dwelling population. *Clin. Interv. Aging.* **2014**, *9*, 79–84.

© 2014 by the authors; licensee MDPI, Basel, Switzerland. This article is an open access article distributed under the terms and conditions of the Creative Commons Attribution license (http://creativecommons.org/licenses/by/4.0/).
